# Ultrastructure of sensilla of antennae and ovipositor of *Sitotroga cerealella* (Lepidoptera: Gelechiidae), and location of female sex pheromone gland

**DOI:** 10.1038/srep40637

**Published:** 2017-01-17

**Authors:** Min Ma, Meng-Meng Chang, Yan Lu, Chao-Liang Lei, Feng-Lian Yang

**Affiliations:** 1 Hubei Insect Resources Utilization and Sustainable Pest Management Key Laboratory, Huazhong Agricultural University, Wuhan 430070, Hubei Province, People’s Republic of China

## Abstract

The Angoumois grain moth, *Sitotroga cerealella*, is a serious pest of stored grains worldwide. Presently, the best effective control against the moth is to disrupt the sexual communication between sexes. Sexual communication in moths includes two processes in which females produce and release pheromones from the sex pheromone gland and males detect and respond to them with a relatively sophisticated olfactory system in their antennae. To better understand these processes, we studied the ultrastructure of antennal and ovipositor sensilla of *S. cerealella* and determined the location of the female sex pheromone gland. Seven types of antennal sensilla were identified on both sexes: sensilla trichodea, sensilla chaetica, sensilla coeloconica, sensilla styloconica, sensilla auricillica, sensilla squamiformia and Bӧhm bristles. Of these sensilla, the sensilla trichodea were significantly more abundant on male antennae than on those of females, suggesting that these sensilla may detect the sex pheromones. On the ovipositor, only sensilla chaetica of various lengths were found. The sexual gland was an eversible sac of glandular epithelium that was situated dorsally in the intersegmental membrane between the 8^th^ and 9^th^ abdominal segments. These results will lead to a better understanding of mate finding with sex pheromones for this worldwide pest species.

The Angoumois grain moth, *Sitotroga cerealella* Olivier (Lepidoptera: Gelechiidae), is one of the most serious stored-grain pests worldwide, particularly in the tropics and warm temperate regions^1^. By feeding inside grains, *S. cerealella* is directly protected from chemical insecticides and causes damage in both the field and storage facilities^2^. Additionally, the increasing use of synthetic insecticides leads to undesirable side effects such as the development of pest resistance and insecticide residues in foods and the environment^3^. Therefore, alternative management tools to control the adult moths are urgently required. In recent years, to combat pest damage, the pest management tactic using behavioural manipulation, including mating disruption, feeding disruption, oviposition deterrence, use of attractants, and pre-release training, has become the focus of research for pest control^4^. Mating disruption, which prevents males from finding females, is the most widely studied area of behavioural manipulation for pest management^4^.

The courtship and copulation of the moth are important behaviours for the pest behavioral manipulation, and the release and reception of sex pheromones regulate the mating behaviours of female and male moths^5^. Female moths emit a volatile pheromone that is detected by males at distance to locate the sexually receptive female, and male antennae have a large number of sensilla that contain olfactory receptor neurons specific to components of the female sex pheromones^6^. The sites for pheromone production and emission are in the terminal abdominal segments of many moth species, including *Cameraria ohridella*^7^, *Helicoverpa zea*^8^ and *Heliothis virescens*^9^. However, the specific site for pheromone production and emission is different among pest species. For example, the sex pheromone gland of *C. ohridella*^7^ is in the dorsal part of the intersegmental membrane between the eighth and ninth abdominal segments, whereas those of *H. zea*^8^ and *H. virescens*^9^ occur as a ring-fold. In *Manduca sexta*, the olfactory sensilla on the antennae are primarily used to identify the female sex pheromone for the location of mates^10^. The similar results were also found in *Agrotis segetum*^11^ and *Cydia pomonella*^12^. For *S. cerealella*, the primary sex pheromone component is Z, E-7, 11-hexadecadien-1-yl acetate^13^, and pheromone-binding proteins of *S. cerealella* postulated to be involved in the detection of sex pheromones are predominantly expressed in adult antennae and display a high binding affinity with Z, E-7, 11-hexadecadien-1-yl acetate^14^. However, the olfactory sensilla and sex pheromone gland of *S. cerealella* have not been fully investigated.

In this study, the external morphology and ultrastructure of the sensilla on the antennae and ovipositor of *S. cerealella* were examined using scanning electron microscopy (SEM) and transmission electron microscopy (TEM) to deduce sensilla function. Additionally, using SEM, histology and gas chromatography-mass spectrometer (GC-MS), the morphology and location of the female sex pheromone gland were determined.

## Results

### General morphology of *S. cerealella* antennae

**Figure 1:**
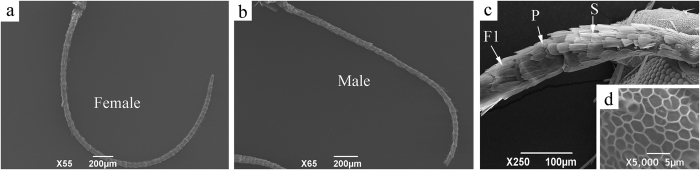
Overview of the general morphology of female and male antennae of *Sitotroga cerealella*. (**a**) Female antenna; (**b**) male antenna; (**c**) base of antenna, showing scape (S), pedicel (P) and the first segment of flagellum (F1); (**d**) the surface of the flagellar segments.

**Figure 2:**
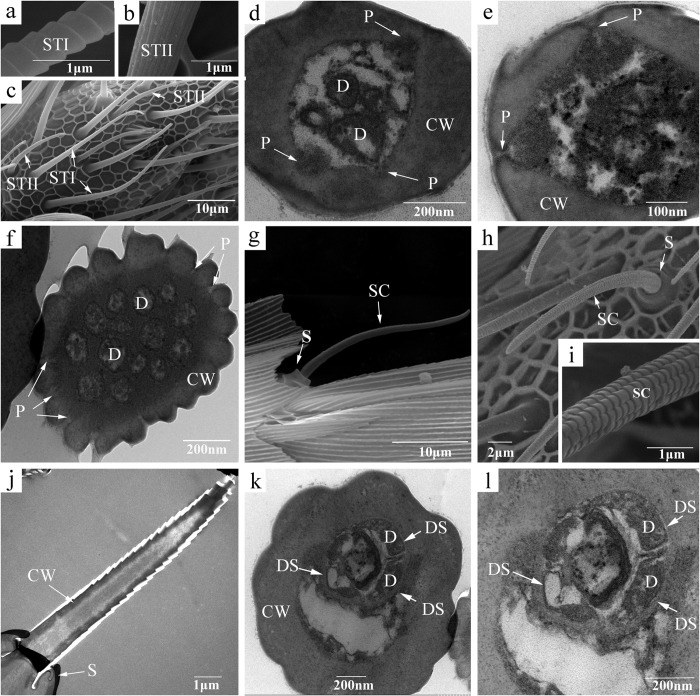
Scanning and transmission electron micrographs of sensilla trichodea (ST) and sensilla chaetica (SC) on the antennae of *Sitotroga cerealella*. (**a,b**) Surface structure of two types of ST, STI (**a**) and STII (**b**); (**c**) shape of STI and STII; (**d,e**) cross sections of STI; (**f**) cross section of STII; (**g,h**) shape of SC; (**i**) surface structure of SC; (**j**) longitudinal section of SC; (**k,l**) cross sections of SC. CW, cuticular wall; P, pores; S, socket; D, dendrites; DS, dendrite sheath.

**Figure 3:**
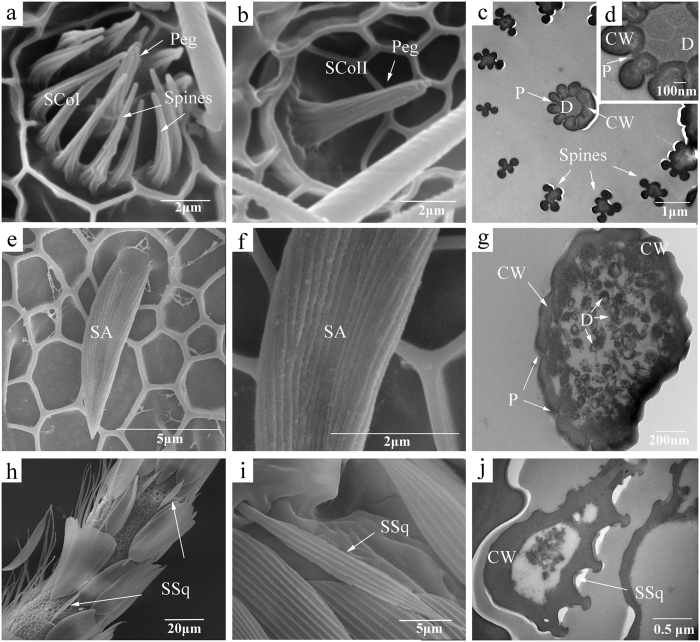
Scanning and transmission electron micrographs of sensilla coeloconica (SCo), sensilla auricillica (SA) and sensilla squamiformia (SSq) on the antennae of *Sitotroga cerealella*. (**a,b**) Shape of two types of SCo, SCoI and SCoII, respectively; (**c,d**) cross sections of SCoI; (**e**) shape of SA; (**f**) surface structure of SA; (**g**) cross section of SA; (**h,i**) shape of SSq; (**j**) cross section of SSq. CW, cuticular wall; D, dendrites; P, pores.

The gross morphology of the antennae of *S. cerealella* was similar between males and females (Fig. 1a,b). The antennae of both sexes were filiform and consisted of three segments: scape, pedicel and a long flagellum composed of 28–34 subsegments (Fig. 1a–c). The length of antennae and the number of flagellar segments flagellomeres were not significantly different between the sexes (P > 0.05; Table 1). The scape (length 269.90 ± 4.31 μm in females and 273.52 ± 5.28 μm in males) was considerably longer than the pedicel (length 79.96 ± 1.66 μm in females and 80.94 ± 2.14 μm in males) (t = 41.109, df = 14.211 and P < 0.001 in females; t = 33.831, df = 14.514 and P < 0.001 in males). The surfaces of both scape and pedicel were smooth and covered with overlapping scales (Fig. 1c). The surface of flagellar segments was a grid or net-like structure (Fig. 1d), and the segments were divided roughly into two primary areas: the dorsal and ventral surfaces. Many types of sensilla were distributed abundantly on the ventral surface. By contrast, the dorsal surface was covered with tile-shaped scales, and only a few sensilla chaetica and sensilla squamiformia located among the scales (Figs 2g and 3h). The length of flagellar segments increased from the base to the apex, whereas the width decreased.

### Different types of antennal sensilla

Seven morphological types of sensilla were observed on both female and male antennae: sensilla trichodea (ST), sensilla chaetica (SC), sensilla coeloconica (SCo), sensilla styloconica (SSt), sensilla auricillica (SA), sensilla squamiformia (SSq), and Bӧhm bristles (BB). The ST, SCo, SSt, and BB also had two subtypes. The length and basal width of these sensilla are shown in Table 2, as are their numbers on each antenna in both sexes. No clear sexual dimorphism occurred between the sexes of *S. cerealella* in the type, size or amount of antennal sensilla except ST. The morphology and distribution of the different sensilla are described in more detail in the following sections.

Sensilla trichodea, found on the ventral and lateral sides of all flagellar segments, were the most abundant type of sensilla on *S. cerealella* antennae. These sensilla were subdivided into two subtypes (STI and STII) according to the surface substructure and the length (Fig. 2a–c). STI were long and slender (length 36.06 ± 2.07 μm in females and 38.21 ± 1.16 μm in males), with annuli or inclined spiral grooves on the surface (Fig. 2a), and were unsocketed at the base and slightly curved towards the apex, forming an angle of 30°–45° between the base of sensilla and the antennal surface (Fig. 2c). No sexual dimorphism was observed for the appearance of STI, but these sensilla were more abundant in males (747.62 ± 20.72) than in females (631.81 ± 20.88, P < 0.01; Table 2). STII were shorter (length 17.47 ± 0.64 μm, t = 8.576, df = 15.462 and P < 0.001 in females; length 19.37 ± 1.14 μm, t = 10.869, df = 27 and P < 0.001 in males) and less abundant (168.90 ± 12.04, t = 19.202, df = 22 and P < 0.001 in females; 170.96 ± 10.72, t = 24.720, df = 16.496 and P < 0.001 in males) than STI, but there was no difference between the sexes. Most of STII were curved at the base and not bent at the end, and the grooves on the sensilla were longitudinal (Fig. 2b,c). Viewed in cross-section, STI had several dendrites in the lumen and thick cuticular wall with a few pores (Fig. 2d,e). The cuticular wall of STII was comparatively thinner and was penetrated by pores, and 8–14 dendrites were observed in the lumen (Fig. 2f).

Sensilla chaetica, with both transversely arranged ridges and sparsely longitudinal grooves on the surface, were similar in shape to STI, but the tips were blunt and sensilla were slightly shorter (length 21.91 ± 0.78 μm in females and 23.97 ± 1.13 μm in males) than the STI (Fig. 2g–i). In addition, the base of the SC was surrounded by a round collar-like socket where was quite different from ST. These sensilla were typically vertical or with a 60° incline towards the antennal surface and were primarily on the ventral and lateral sides of the flagella, but one or two SC were on the middle part of the dorsal side of each flagellar segment. For SC, no differences between the sexes were found. In the TEM micrographs, these sensilla had thick cuticular wall with no pores, and the distal dendrites were enveloped in a dendrite sheath (Fig. 2j–l).

Sensilla coeloconica were on the ventral side of the flagellum, and two subtypes were identified: SCoI and SCoII. SCoI had a flower-like shape with a peg in the center, which was surrounded by approximately 11–14 inwards-facing spines that resembled petals (Fig. 3a). The cuticular surface of these sensilla had longitudinal striations. Except for the terminal segment, each flagellar segment possessed approximately 1–4 SCoI. The differences between the sexes for basal width and numbers of sensilla were not significant (P > 0.05; Table 2). Similar to SCoI, SCoII had the peg in the centre of the pit but were without the spines (Fig. 3b). This sensilla subtype was rare and was found only occasionally on individual antenna. The SCo were double-walled sensilla and had longitudinally arranged slit-like pores (Fig. 3c,d). Transverse sections showed about four dendrites in the lumen of the sensilla (Fig. 3c,d).

Sensilla auricillica had the appearance of a rabbit ear or a new leaf of Gramineae (Fig. 3e). The cuticular surface had longitudinal grooves, and these sensilla were bent towards the apex of the antenna and were almost parallel to the antenna surface (Fig. 3e,f). The SA were similar between sexes in length, basal width, and numbers (P > 0.05; Table 2). In the TEM micrograph of the cross section, the cuticle wall was extremely thin and penetrated by a dense abundance of pores, with numerous distal dendrites in the lymph (Fig. 3g).

Sensilla squamiformia were distributed on the dorsal surface of all parts of the antenna and were covered with scales. These sensilla were scale-like, but were smaller and narrower than normal scales (Fig. 3h,i). The appearance and number of SSq were similar between sexes (P > 0.05; Table 2). The cuticle of these sensilla had longitudinally arranged furrows, but no wall pores was found (Fig. 3j).

**Figure 4:**
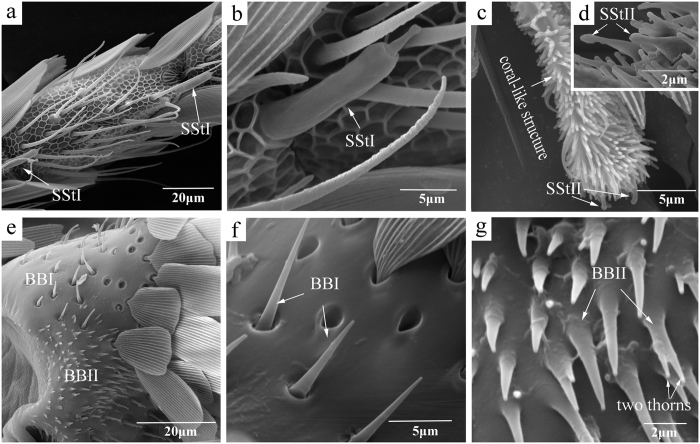
Scanning electron micrographs of sensilla styloconica (SSt) and Böhm bristles (BB) on the antennae of *Sitotroga cerealella*. (**a–d**) Shape of two types of SSt (SStI and SStII); (**e–g**) shape of two types of BB (BBI and BBII).

Sensilla styloconica were divided into two subtypes, SStI and SStII, based on specific features. SStI were on the distal margin of each flagellomere with the exception of the apical segment, and there was only one sensillum on each segment (Fig. 4a,b). This type of sensilla was stout and thumb-like at the base with one cone-shaped structure apically, which swelled slightly into a spherule at the top (Fig. 4b). The surface of these sensilla was smooth, but some cuticular ridges were found at the base. These sensilla were approximately 15 μm in length (14.98 ± 1.27 μm in females and 16.93 ± 0.46 μm in males) and had a basal width of approximately 3.5 μm (3.58 ± 0.15 μm in females and 3.54 ± 0.10 μm in males; Table 2). No significant differences were found between the sexes (P > 0.05). SStII were only found on the terminal segment of the antenna where there was a coral-like structure that densely covered with blunt short hairs rather than scales (Fig. 4c,d). Two SStII grew out side by side from the end of the coral-like structure.

The short spines of Bӧhm bristles are classified by Schneider^15^ as sensilla chaetica, but these bristles were shorter and sharper than sensilla chaetica and were distributed in clusters on the bases of scape and pedicel segments (Fig. 4e–g). Based on morphological characteristics, BBs were divided into two subtypes: BBI and BBII. BBI, with a smooth cuticle, sat upright from sockets on the antenna surface, whereas BBII, without sockets, emerged directly from the cuticle and were found only on the base of the scape (Fig. 4e–g). Additionally, the surface of the base of BBII was not smooth, with some even separated into two thorns near the apex (Fig. 4g). Because BB on the pedicel segments were always covered by scales, these sensilla were not counted.

### Morphology of the extruded terminal abdominal segments of female *S. cerealella*

**Figure 5:**
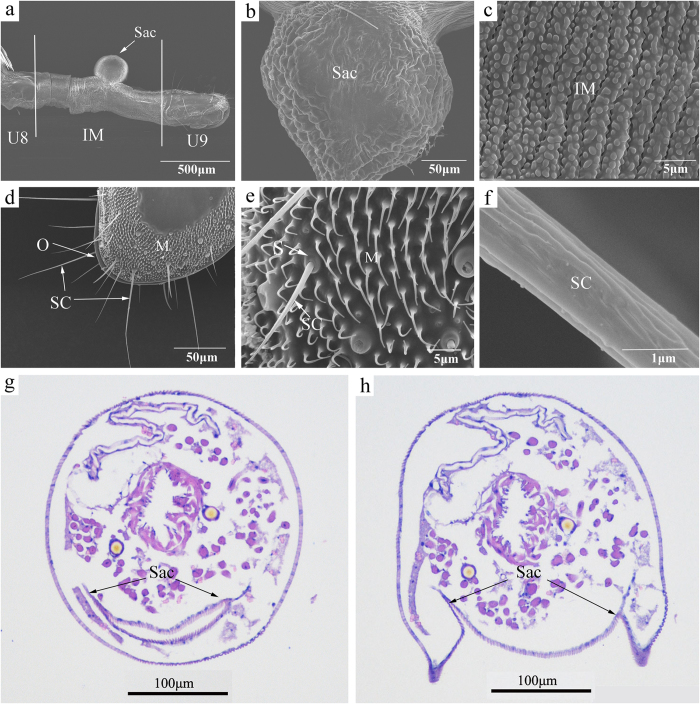
Terminal portion of abdomnden with ovipositor and pheromone gland from female *Sitotroga cerealella*. (**a**) Overview of extruded terminal abdominal segments of female *S. cerealella* under scanning electron microscopy; (**b**) eversible sac, showing the wrinkles on the surface; (**c**) surface structure of 8^th^–9^th^ intersegmental membrane, showing the conspicuous wrinkles and granular protuberances; (**d,e**) ovipositor sensilla of *S. cerealella*, showing the features of sensilla chaetica and microtrichia; (**f**) surface structure of SC; (**g,h**) histological sections through the glandular region with the sac in retracted position and the sac bulged out, respectively. Purple indicates cytoplasm, and blue represents cell nucleus. U8, 8^th^ uromere; U9, 9^th^ uromere; IM, intersegmental membrane of 8^th^–9^th^ uromeres; O, ovipore; SC, sensilla chaetica; S, socket; M, microtrichia.

The 7^th^ abdominal segment of female *S. cerealella* was densely clothed with scales; whereas the 8^th^ and 9^th^ uromeres were without scales (Fig. 5a). The 9^th^ uromere was connected to the 8^th^ uromere by a sleeve-like membrane (Fig. 5a). According to the description of the sex *pheromone* gland of the gelechiid moth *Pectinophora gossypiella*, the pink bollworm^16^, we speculated that the eversible sac situated dorsally in the 8^th^–9^th^ intersegmental membrane of *S. cerealella* was the gland for producing the sex pheromone (Fig. 5a,b). The surface of the sac was uneven (Fig. 5b). The surface of the intersegmental membrane was almost completely covered with a continuous series of conspicuous granular protuberances and irregular, deep grooves (Fig. 5c). The 8^th^ and 9^th^ uromeres of female *S. cerealella* were normally folded and embedded within the 7^th^ segment.

Only sensilla chaetica (SC) and microtrichia were found on the ovipositor of *S. cerealella*. SC were of varying lengths and were sparsely distributed on the edge of the ovipositor, particularly on the two sides of the ovipore region (Fig. 5d). These sensilla were set in tight sockets, similar to SC on the antennae, but were much sharper than the latter (Fig. 2g,h). Microtrichia were small hairs that protruded from the surface of the ovipositor, and densely covered on the two lateral sides of the ovipositor. Some microtrichia were straight or slightly curved towards apex; whereas others curved hook-like, with the microtrichia more curved closer to the ovipore region (Fig. 5d,e).

### Histology of female sex pheromone gland

The eversible sac remained folded into the intersegmental membrane when we did not extrude the abdomen of the llive moth (Fig. 5g). With the ovipositor extended, the 8^th^ and 9^th^ segments were fully exposed (Fig. 5h). The gland cells of *S. cerealella* were the identical size and shape as the epidermal cells of the intersegmental membrane, so the limits of the sex pheromone gland could not be determined using histological methods.

### Component and location of female sex pheromone gland determined with GC-MS

**Figure 6:**
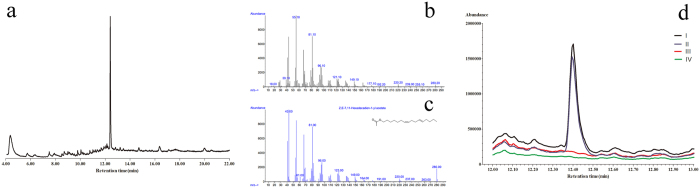
Chromatogram and mass spectra of abdominal extracts of female *Sitotroga cerealella*. (**a**) Total ionic chromatogram of the components from the abdominal extracts of female *S. cerealella*; (**b**) Mass spectra at retention time 12.4 min of female gland extracts; (**c**) standard mass spectrum of Z, E-7, 11-hexadecadien-1-yl acetate (sex pheromone of *S. cerealella*) from the National Institute of Standards and Technology library; (**d**) Chromatogram of abdominal extracts of female *S. cerealella*: I, terminal portion of abdomen; II, intersegmental membrane of 8^th^**–**9^th^ uromeres; III, 8^th^ uromere; IV, 9^th^ uromere.

The GC-MS results showed that the sex pheromone had only one primary component, composing 85% of the entire volatile fraction at the retention time of 12.4 min (Fig. 6a). The confirmation of the analytes was based on the retention times and the presence of one fragment ion for the compound. Both retention time and relative abundance of the diagnostic ions were required to be within established ranges. The precursor ions selected for the compound were the characteristics of m/z for Z, E-7, 11-hexadecadien-l-yl acetate from the results of mass spectra for female gland extracts at a retention time of 12.4 min and those of the standard mass spectrum of Z, E-7, 11-hexadecadien-l-yl acetate in the National Institute of Standards and Technology library, with a similarity that reached 99% (Fig. 6b,c). Therefore, 7Z, 11E-hexadecadien-1-ol acetate was confirmed as the component at the retention time of 12.4 min. To detect the specific location of the female sex gland that released sex pheromone, extracts from the terminal portion of the female abdomen were analyzed, i.e., the 8^th^ and 9^th^ abdominal segments and the intersegmental membrane between the 8^th^ and 9^th^ segments. Only the intersegmental membrane between the 8^th^ and 9^th^ abdominal segments released the component with one peak at the retention time of 12.4 min, whereas the other sections did not; thus, the sex pheromone was released from the intersegmental membrane between the 8^th^ and 9^th^ segments (Fig. 6d).

## Discussion

Sexual communication plays an important role in the mating behaviours of moths for reproduction^17^. Communication between sexes is a two-way process: the females produce and release pheromone from the sex pheromone gland, which is generally located on the terminal abdominal segments^18^, and then the males detect and respond to these sex pheromones from a distance^19^. Antennae are the primary sense organs in insects^15^. Most of the olfactory organs are located on the antennal flagellum and often take the form of microscopic hairs called sensilla^20^. In this study, we focused on the sensilla of the antennae and ovipositor of *S. cerealella* and the location of the female sex pheromone gland, which are the structures most likely involved in pheromone communication.

Generally, sensilla with pores distributed over the cuticular walls are involved in chemoreception^21^. In this study, seven different types of sensilla were identified on the antennae of *S. cerealella*. Of these sensilla, three have pores in the cuticular walls, i.e., sensilla trichodea, sensilla coeloconica and sensilla auricillica, suggesting that these sensilla may be associated with olfaction of the moth.

Sensilla trichodea, divided into two subtypes, were the most widespread and numerous sensilla on the antennae of *S. cerealella*. These sensilla are widely believed to be olfactory receptors^22^. STI were significantly more abundant in males than in females, which is similar to the descriptions for *P. gossypiella*^23^ and *Ostrinia nubilalis*^24^. The function of STI may explain the sexual dimorphism. Many studies show that STI with well-developed pores in thick cuticular walls likely contain olfactory receptors that detect the sex pheromones^10^,^25^. The function of STII remains unclear; however, STII of *Zamagiria dixolophella* Dyar may be used for sex pheromone detection^26^.

For sensilla coeloconica of *S. cerealella*, two subtypes were observed, one with spines and one without, which were identical to those in *M. sexta*^27^, *Mythimna separata*^28^ and *Loxostege sticticalis*^29^. These double-walled and multiporous sensilla could be exclusively olfactory^27^. However, the spines outside the peg are suggested to protect the peg from physical damage by the environment^30^. These sensilla are recessed in the antennal surface. This is not optimal for receiving chemical stimuli, but is likely a necessary adaptation to prevent desiccation^31^. SCo may also participate in the processes of hygro- and thermoreception^32^ and the perception of CO_2_^33^.

The ear-like sensilla auricillica described for *S. cerealella* in this study were similar to those reported for *L. sticticalis*^29^, *Cnaphalocrocis medinalis*^34^ and *Scoliopteryx libatrix*^35^. These sensilla were primarily characterized by the extremely thin cuticular wall penetrated by a dense pattern of pores, with numerous distal dendrites in the lymph. Some researchers suggest that this type of sensillum may be preferentially receptive to plant volatiles^35^, whereas SA of *C. pomonella* may respond to potential minor sex pheromone components^12^. Therefore, the function of these sensilla in *S. cerealella* might be involved in odor recognition. Further investigations, such as electrophysiological and behavioural experiments, are necessary to elucidate its function.

The cuticular specializations on the ovipositor of *S. cerealella* were similar to those found on the European corn borer, *O. nubilalis*^36^. Ovipositor sensilla of many species have both mechanosensory and chemosensory functions^37^. Sensilla chaetica of varying lengths were the only type of sensilla on the ovipositor of *S. cerealella*. In female *H. virescens*, these hairs have a possible chemosensory function for pheromones^38^. However, similar sensilla in *Bombyx mori* have a mechanosensory function as demonstrated by electrophysiology^39^. Microtrichia are small hairs without a socket that may be simple cuticular ornamentations^36^ and common on ovipositors in other Lepidoptera^37^,^40^.

Glands of female Lepidoptera are most commonly found as modifications of the intersegmental membrane between the 8^th^ and 9^th^ abdominal segments^41^, which is a location mostly consistent with that in other orders^42^; however, the exact location of where the membrane becomes glandular is extremely variable among female lepidopterans^42^. According to the description of the sex pheromone gland of other gelechiid moth *P. gossypiella*^16^ and *Phthorimaca operculella*^43^, we speculated that the eversible sac situated dorsally in the 8^th^–9^th^ intersegmental membrane of *S. cerealella* was the sex pheromone gland of female moth. Similar structures are also found in moths of other families such as *C. ohridella*^7^ and *O. nubilalis*^44^. In addition, these scent folds may also occur on the ventral body side as in *Prodenia ornithogalli*^9^ and *Yponomeuta latreille*^45^ or as a ring-fold in *H. zea*^8^, *Heliothis phloxiphaga* and *H. virescens*^9^. The sex pheromone of *S. cerealella* is assumed to diffuse through the cuticular surface of the sac because there are no pores or other gland openings in the cuticle of the gland. Additionally, the granular protuberances and deep grooves on the surface of intersegmental membrane enlarge the surface area of the pheromone gland, which may facilitate dissemination of pheromones from the gland, as occurs in *C. ohridella*^7^.

The gland cells are hypertrophied epidermal cells in the intersegmental membrane between the 8^th^ and 9^th^ abdominal segments^42^, as previously demonstrated in *Choristoneura fumiferana*^46^ and *C. ohridella*^7^. However, with the histological methods used in this study, the gland cells of *S. cerealella* were the identical size and shape as the epidermal cells of the intersegmental membrane. A similar phenomenon was observed with *P. gossypiella*^16^. So the specific dimensions of the pheromone gland could not be determined using histological methods.

The location of the sex pheromone-producing gland in female *S. cerealella* was further determined by GC-MS analysis of female sex pheromone extracts. As reported previously, the primary component of the sex pheromone was identified as Z, E-7, 11-hexadecadien-l-yl acetate^13^. Based on a comparison of the individual extracts, we further defined that the sex pheromone gland was on the intersegmental membrane between the 8^th^ and 9^th^ abdominal segments. Additionally, according to our practical investigations, females of *S. cerealella* exhibit a characteristic calling behaviour soon after eclosion, and become quiescent and protrude the terminal abdominal segments (8^th^ and 9^th^ abdominal segments). The females remain in this position for several hours to attract males. These results further verified that the terminal abdominal segments (8^th^ and 9^th^ abdominal segments) were the source of the female sex pheromone in *S. cerealella*.

In this study, the morphology, fine structures and distributions of the sensilla on the antennae and ovipositor of *S. cerealella* were analyzed, and the structure and location of the female sex pheromone gland were also determined. These analyses allow us to better understand the olfactory mechanisms for chemical communication between males and females and provide a useful foundation for future electrophysiological and behavioural studies.

## Materials and Methods

### Insects

Adult *S. cerealella* were obtained from a laboratory colony that was originally collected from Wuhan, Hubei Province, China, in July 2010. The moths were maintained in cages (25 × 25 × 25 cm) containing an approximately 3-cm-thick layer of wheat on the bottom. To obtain virgin adults, each kernel that contained one pupa was separated in a small glass tube, which were maintained at 28 ± 1 °C, 75% ± 5% relative humidity and a photoperiod cycle of 14 h light/10 h dark. The pupae were checked daily until eclosion, and the newly emerged adults were used in the experiments.

### Electron microscopy

Adults of *S. cerealella* were first anaesthetized by aether. Then, the antennae and extended ovipositors (by applying slight pressure on the abdomen, Fig. 5a) were dissected and immersed in glutaraldehyde (2.5%) at 4 °C for 24 h. After washing in phosphate-buffered saline (PBS, 0.1 M, pH = 7.4) 2–3 times (10 min/wash), samples were post-fixed in 1% osmium tetroxide in PBS at 4 °C for 2 h and then dehydrated in a graded series of ethanol (30%, 50%, 70%, 80%, 95%, and twice at 100%, each for 15 mins).

For scanning electron microscopy, samples were substituted in ethanol-isoamyl acetate (1:1 and 1:2, v/v) and finally immersed in 100% isoamyl acetate for drying in a critical point drier (Hitachi HCP-2, Hitachi, Tokyo, Japan). After mounting on a holder using double-sided adhesive tape, all samples were sputter-coated with gold/palladium (40:60) and observed using a Hitachi jsm-6390 or SU8000 scanning electron microscope.

For transmission electron microscopy, the antennae were embedded in SPI-812 resin. Ultrathin sections were cut with a diamond knife on a Leica UC6 ultramicrotome, double-stained with uranyl acetate and lead citrate and finally observed on a Hitachi HT7700 transmission electron microscope operating.

### Histology

The ovipositors from females were fixed in 4% paraformaldehyde for 24 h. After fixing, samples were dehydrated in a graded series of ethanol: 75% for 4 h, 85% for 2 h, 90% for 2 h, 95% for 1 h, and twice at 100% for 30 min each. The specimens embedded in wax were then serial sectioned. The slices, 4 μm thick, with sagittal and cross sections, were stained with hematoxylin-eosin stain and later dehydrated. Finally, rhamsan gum was used to seal the slices, with the histology sections examined with a Nikon Eclipse Ti-SR microscope (Nikon, Tokyo, Japan).

### Extraction of female gland extracts

Pressure was applied to the abdomen of virgin female moths (2-d-old) until the terminal abdominal segments extruded. Ovipositors (8^th^ and 9^th^ abdominal segments) were then excised from twenty virgin females and extracted in 100 μl of hexane for 30 min. The extracts were analyzed using GC-MS. Another twenty ovipositors from 2-d-old scotophase females were separately cut into three sections (U8, IM and U9) as shown in Fig. 5a, with each section individually extracted and analyzed for pheromone with GC-MS.

### Detection of female gland extracts

The gland extracts were analyzed using a DB-WAX capillary column (30 m length, 0.25 mm id, 0.25 μm film thickness) with a GC-MS system (7890A-5975C). The inlet (250 °C) was operated in splitless mode (7 psi, He, constant pressure) and 3 μl of the sample was injected (MS solvent delay 4 min). The oven was operated as follows: 50 °C for three minutes to 230 °C for ten minutes at a rate of 20 °C/min. Helium carrier gas was used at a constant flow rate of 1.0 ml/min. Electron impact mass spectra were recorded at 70 eV ionization energy in full scan mode. The ionization source temperature was set at 230 °C.

### Terminology and Data Process

The morphological terms in this study followed those of Schneider^15^, Zacharuk^47^ and Keil^48^. The differences in number and size of antennal sensilla between males and females were analyzed using an independent-sample t-test (P < 0.05) with the SPSS statistical software package version 19.0.

## Additional Information

**How to cite this article**: Ma, M. *et al*. Ultrastructure of sensilla of antennae and ovipositor of *Sitotroga cerealella* (Lepidoptera: Gelechiidae), and location of female sex pheromone gland. *Sci. Rep.* **7**, 40637; doi: 10.1038/srep40637 (2017).

**Publisher's note:** Springer Nature remains neutral with regard to jurisdictional claims in published maps and institutional affiliations.

## Figures and Tables

**Table 1:** General characteristics of the antennae of *Sitotroga cerealella.*

Gender	Antenna length (μm)	Scape length (μm)	Pedicel length (μm)	No. of flagellomere
♀	3198.09 ± 125.39	269.90 ± 4.31	79.96 ± 1.66	31.25 ± 0.45
♂	3204.05 ± 135.76	273.52 ± 5.28	80.94 ± 2.14	31.17 ± 0.41

Values are presented as the means ± s.e.m. Data comparing sexes in each column are not significantly different (P > 0.05; independent-sample t-test). N = 12 per sex.

**Table 2:** Sizes and numbers of antennal sensilla on male and female *Sitotroga cerealella*.

Sensilla type	Gender	Length (μm)	Basal width (μm)	No. of sensilla
STI	♀	36.06 ± 2.07	1.85 ± 0.05	631.81 ± 20.88
	♂	38.21 ± 1.16	1.87 ± 0.03	747.62 ± 20.72**
STII	♀	17.47 ± 0.64	1.87 ± 0.04	168.90 ± 12.04
	♂	19.37 ± 1.14	1.91 ± 0.05	170.96 ± 10.72
SB	♀	21.91 ± 0.78	1.70 ± 0.04	143.60 ± 6.92
	♂	23.97 ± 1.13	1.72 ± 0.06	133.19 ± 7.84
SCoI	♀	—	6.65 ± 0.18	72.6 ± 6.36
	♂	—	6.79 ± 0.09	73.92 ± 6.37
SCoII	♀	—	—	—
	♂	—	—	—
SStI	♀	14.98 ± 1.27	3.58 ± 0.15	32.25 ± 0.45
	♂	16.93 ± 0.46	3.54 ± 0.10	32.17 ± 0.41
SStII	♀	—	—	—
	♂	—	—	—
SA	♀	10.15 ± 0.78	1.84 ± 0.08	45.38 ± 4.56
	♂	10.34 ± 0.17	1.77 ± 0.09	47.77 ± 5.82
SSq	♀	22.69 ± 0.75	1.30 ± 0.09	73.54 ± 5.04
	♂	21.61 ± 0.44	1.17 ± 0.04	83.42 ± 5.87
BBI	♀	6.22 ± 0.24	0.99 ± 0.04	—
	♂	6.94 ± 0.40	1.10 ± 0.38	—
BBII	♀	—	—	—
	♂	—	—	—

Values are presented as the means ± s.e.m. The difference between the means for the sexes in each column followed by ** is significant (P < 0.01; independent-sample t-test). N = 12 per type of sensilla. “—” means that data are not measured.
